# Harnessing rhizospheric core microbiomes from arid regions for enhancing date palm resilience to climate change effects

**DOI:** 10.3389/fmicb.2024.1362722

**Published:** 2024-04-05

**Authors:** Ameni Ben Zineb, Mariem Lamine, Ahlem Khallef, Helmi Hamdi, Talaat Ahmed, Hareb Al-Jabri, Mohammed Alsafran, Ahmed Mliki, Sami Sayadi, Mahmoud Gargouri

**Affiliations:** ^1^Center for Sustainable Development, College of Arts and Sciences, Qatar University, Doha, Qatar; ^2^Laboratory of Plant Molecular Physiology, Centre of Biotechnology of Borj-Cedria, Hammam-Lif, Tunisia; ^3^Higher Institute of Biotechnology of Monastir, University of Monastir, Monastir, Tunisia; ^4^Environmental Science Center, Qatar University, Doha, Qatar; ^5^Department of Biological and Environmental Sciences, College of Arts and Sciences, Qatar University, Doha, Qatar; ^6^Agricultural Research Station, Office of VP for Research and Graduate Studies, Qatar University, Doha, Qatar

**Keywords:** date palm, desert plant microbes, sustainability, biofertilizers, GCC

## Abstract

Date palm cultivation has thrived in the Gulf Cooperation Council region since ancient times, where it represents a vital sector in agricultural and socio-economic development. However, climate change conditions prevailing for decades in this area, next to rarefication of rain, hot temperatures, intense evapotranspiration, rise of sea level, salinization of groundwater, and intensification of cultivation, contributed to increase salinity in the soil as well as in irrigation water and to seriously threaten date palm cultivation sustainability. There are also growing concerns about soil erosion and its repercussions on date palm oases. While several reviews have reported on solutions to sustain date productivity, including genetic selection of suitable cultivars for the local harsh environmental conditions and the implementation of efficient management practices, no systematic review of the desertic plants’ below-ground microbial communities and their potential contributions to date palm adaptation to climate change has been reported yet. Indeed, desert microorganisms are expected to address critical agricultural challenges and economic issues. Therefore, the primary objectives of the present critical review are to (1) analyze and synthesize current knowledge and scientific advances on desert plant-associated microorganisms, (2) review and summarize the impacts of their application on date palm, and (3) identify possible gaps and suggest relevant guidance for desert plant microbes’ inoculation approach to sustain date palm cultivation within the Gulf Cooperation Council in general and in Qatar in particular.

## 1 Introduction

Among the arid zones of the globe, which occupy a third of the Earth’s surface (Neilson et al., 2017), the Gulf Cooperation Council (GCC) region, known for its large sandy deserts (Alsharif et al., 2020), has experienced the most intense climate changes over the last two decades, inducing profound disruptions that seriously threaten the sustainability of existing agrosystems (Fernández-López et al., 2022).

Within this area, the cultivation of date palm (*Phoenix dactylifera* L.), or phœniciculture, is the flagship activity, the symbol of life, and the cornerstone of oasis agrosystems. It is considered as a “holy tree” due to its vital nutritional (fruits and by-products) and socio-economic importance (Almadini et al., 2021; Naqvi et al., 2021). In addition to its agronomic and economic importance, date palm is known to tolerate harsh environmental conditions such as extreme temperature, drought, and high salinity of soil (Abumaali et al., 2023b). Since the Middle East has sandy soils and a dry climate, this tree contributes efficiently to mitigating desertification and erosion and preserving the oasis microclimate (Jain et al., 2011). Nevertheless, and despite this robustness, date palm encounters diverse constraints that, if no intelligent strategies are urgently implemented, would progressively compromise its sustainability and, in the long term, its extinction from this geographical area (El-juhany, 2014; Almadini et al., 2021). Among these structural and climatic constraints are: low organic soil matter content (Darwish and Fadel, 2017), limited and reduced groundwater levels (Sherif et al., 2023), growing salinity (Al Kharusi et al., 2019), resurgence and spread of pests and diseases (Alotaibi et al., 2023), and a very limited survival rate of newly planted plantlets due to their low potential to adapt to the harsh conditions of the environment (Saadaoui et al., 2019). Indeed, date palms are adapted to arid and semi-arid regions. They withstand moderately alkaline soils, with a pH ranging from 7 to 8.5, but extremely high alkalinity can be detrimental to their growth (Alotaibi et al., 2023; Sanka Loganathachetti and Mundra, 2023). They are adapted to soils with low organic matter levels, ranging from 0.1 to 1 (Mlih et al., 2016, 2019), while proper fertilization is essential to ensure their optimal growth. Furthermore, date palms exhibit a notable tolerance to salinity levels, commonly ranging from 4 to 8 dS/m or even higher under specific conditions (Muller et al., 2017; Alotaibi et al., 2023). Nevertheless, the irrigation of date palm with saline groundwater, due to water scarcity, is a common practice in the MENA regions, which threatens the sustainability of date palm cultivation (Shamim et al., 2022; Sanka Loganathachetti and Mundra, 2023).

To overcome these constraints, several useful solutions have been developed (Cai and Liu, 2015), among them, biotechnological approaches involve innovative breeding programs (Hazzouri et al., 2020). However, the efficiency of these conventional approaches in combating various stresses in plants was limited on one hand, and the non-conventional approaches, such as tissue *in vitro* technology, require more labor and may produce less resilient plants, particularly in woody trees (Al-Khateeb et al., 2020). Moreover, the excess of chemical fertilizer amendments and agricultural practices (e.g., tillage) has dramatically affected the diversity of the beneficial soil microbiota. This situation is worsened by intense climate changes, which impart severe impacts on plant–soil–microorganism interactions by altering the structure, abundance, composition, and functional activity of the rhizosphere microbiome (Sabir et al., 2021). Indeed, plants’ endosphere, rhizosphere, leaves, and other tissues are home to a multitude of microorganisms, known as the microbiome (Bonatelli et al., 2021). The rhizosphere microbiome interacts with and affects, often positively, the adaptation of its host plants to their environment (Bais et al., 2006; Kumar and Dubey, 2020). Indeed, the influence of plant roots on microbes is governed by the root exudates (Williams and de Vries, 2020), which include low-molecular-weight primary metabolites, like organic acids, amino acids, and sugars, and secondary metabolites, such as phenols, flavonoids, and terpenoids (De Vries et al., 2019). A plant’s exudate may be affected by climate change as a result of alterations in the plant’s photosynthetic apparatus, which will indirectly affect the root microbes by changing the carbon sources available to them and leading to their cell lysis (Chen et al., 2022). In their study, Naylor et al. (2017) compared the root rhizosphere of 18 species of monocot plants under drought stress and found that *Actinobacteria* are more abundant during a water deficit. Rice root-associated microbiota were also found to be enriched in Actinobacteria and *Chloroflexi* under drought stress, while several *Acidobacteria* and *Deltaproteobacteria* were depleted (Andreo-Jimenez et al., 2019).

Several ecosystem processes are directly or indirectly influenced by soil microorganisms, which play a vital role in enhancing ecosystem resilience and complexity (Robinson et al., 2023). These microorganisms are known to have beneficial attributes promoting nutrient cycling (e.g., solubilizing or decomposing soil’s below-ground complexed phosphorus, Ben Zineb et al., 2019b), plant health (e.g., systemic tolerance can be induced by plant growth-promoting microorganisms through biochemical mechanisms) (De Zelicourt et al., 2013), and climate regulation (e.g., CO_2_, CH_4_, and N_2_O producing or consuming). Therefore, their use in the restoration, conservation, and maintenance of the date palm ecology and production is becoming more challenging. There is an urgent need for more integrated research to improve simultaneously the productivity of the low-cost date palm system and its sustainability and to develop technologies favoring/restoring its microbial diversity.

Microbes play crucial roles in the rhizosphere of date palm, contributing to its overall health and nutrient availability. Indeed, several investigations have uncovered a wide spectrum of interactions between plant growth-promoting (PGP) microbes and date palm, including the promotion of shoot and root growth (Cherif et al., 2015), inducing systemic tolerance against abiotic stresses (Harkousse et al., 2021), as well as the inhibition of some pathogenic fungi (Siala et al., 2016). In light of this, date palm sustainability can be met through the intelligent use of native plant microbiomes, which boost plant potential to adapt and survive under intense abiotic stresses (Koziol et al., 2018; Qiu et al., 2019; Alsharif et al., 2020). Among these, desert indigenous microorganisms are increasingly recognized as a long-term environmental and ecological potential solution to sustain agriculture in the oasis ecosystem (Almutawa, 2022). Their application benefits have been documented to: (i) promote date palm growth and survival rate of seedlings in the nursery (Shabbir et al., 2011); (ii) improve nutrient uptake by maintaining metabolic processes (Van Oosten et al., 2017; Alsharif et al., 2020); (iii) improve resistance to harmful pathogens (Mefteh et al., 2018); and (iv) induce better tolerance to complex abiotic stresses, drought, and salinity at a priority level (Benhiba et al., 2015; Akensous et al., 2022b). Consequently, focusing on beneficial microorganisms from arid and desertic lands would represent potential and innovating biotechnological tools to restore and promote agricultural activity in desertic areas in general and of date palm in oases in particular (Symanczik et al., 2014; Ferjani et al., 2015).

Alotaibi et al. (2023) reported on date palm biotechnology, including overviews of soil and environmental conditions of date palm cultivation, and Jain et al. (2011) reported on research progress and applications in this domain. Nevertheless, no holistic and comprehensive review has yet been conducted on the arid land’s microbiome, including prokaryotic and fungal communities, and its contribution to phœniciculture sustainability. Therefore, a deep study of the diversity and role of the date palm microbiome, including bacteria, fungi, archaea, viruses, and other microbes, is particularly relevant. Decades of research have demonstrated the importance of gathering information from the genetic repertoires of microbial communities from various hosts (Lucaciu et al., 2019). The recent advent of high-throughput sequencing technologies coupled with a variety of “omics” techniques has marked the beginning of a new green era in agriculture (Pantigoso et al., 2023). Modern sequencing techniques, such as next-generation sequencing, 16S rRNA gene sequencing, internal transcribed spacer sequencing, or the combination of these methods, provide in-depth information about plant microbial partners. They were able to better characterize the structure and function of these communities (Mitter et al., 2019; Satam et al., 2023). Still, the cultivation of those microbial partners is needed. Indeed, engineering date palm cultivable microbes might involve two approaches, either by re-introducing *in situ* enriched indigenous beneficial microorganisms or by inoculating them with exogenous beneficial microorganisms. *In situ* direct inoculation of microbes with PGP activities is the most commonly used strategy to enhance date palm growth (Yaish et al., 2015; Hazzouri et al., 2020). Although inoculating date palm with exogenous microorganisms may not directly promote their growth, they could still benefit by recruiting other microbial species able to enhance their resilience against abiotic stress (Alsharif et al., 2020).

Along with the incessant search for sustainable processes to produce dates in hyper-arid ecosystems, like the GCC area, there is a need to collect updated information, encouraging researchers to engage in new eco-friendly, insightful studies. Therefore, this review critically reports knowledge and pertinent scientific achievements on desert plant-associated microorganisms and their applications on date palms. We also targeted the knowledge available, the gaps, and what would be recommended for the desert plant microbes’ inoculation approaches to sustain the GCC phœniciculture, with a particular emphasis on Qatar.

## 2 Desert plant-associated microorganism: a reservoir of efficient biofertilizers

Decades of empirical and theoretical research have revealed that plants are not standalone entities. They are influenced by their association with microbiota, named “holobiont” (Vandenkoornhuyse et al., 2015; Wagg et al., 2022). The holobiont of desert plants is the center of interest regarding its performance under severe environmental constraints (Araya et al., 2020). Nowadays, several projects have reported promising results for the improvement of agricultural production systems sustainability, owing to inoculation with microbial rhizospheres deriving from plants surviving in arid and desertic areas (Köberl et al., 2011, 2013; Eida et al., 2018; Ha et al., 2021; Procter et al., 2022).

**Figure 1:**
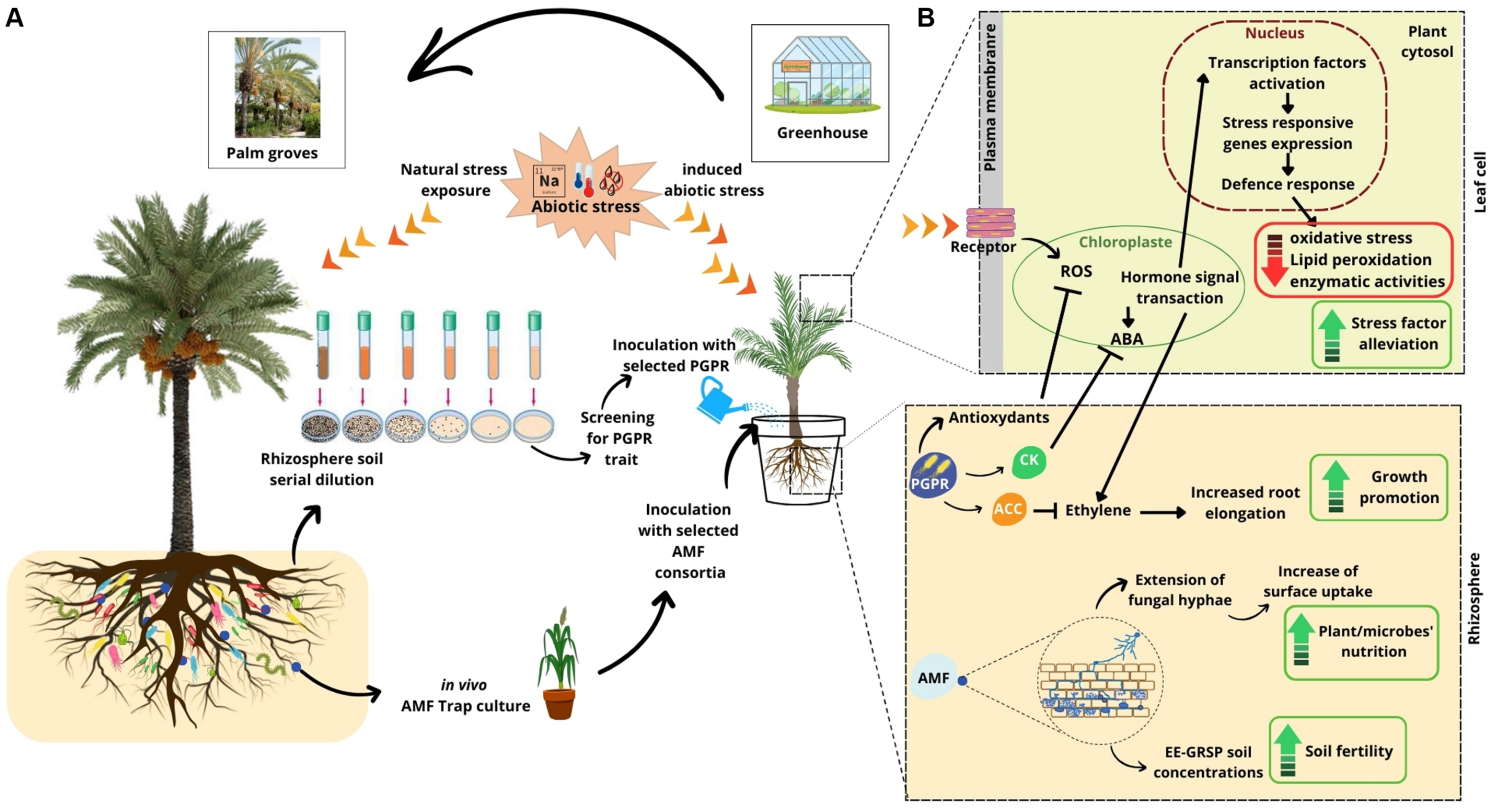
**(A)** An overview of the date palm close-up inoculation system through re-introduction *in situ* of enriched indigenous beneficial microorganisms. **(B)** Date palm perception of external stimuli and the activation of both direct and indirect defense mechanisms to support the plant in dealing with abiotic stresses. Arrows indicate promotion; blunt-ended lines indicate inhibition. All the stress response mechanisms of PGP bacteria and AMF shown in this figure are synthesized from the studies listed in Table 1. AMF, arbuscular mycorrhizal fungi; PGP, plant growth-promoting; IAA, indole-3-acetic acid; ABA, abscisic acid; ACC, 1-aminocyclopropane-1-carboxylic acid; CK, cytokinin; ROS, reactive oxygen species.

This high-performance potential was reported for the holobiont of the desert plant cassava (*Manihot esculenta* Crantz) (Ha et al., 2021). The African desert grass *Stipagrostis pungens*, grown under severe drought conditions, revealed harboring beneficial bacteria that produce extracellular polymeric substances (e.g., exopolysaccharide), which form a hydrophilic biofilm around plant roots (Rolli et al., 2015). Thus, protecting the roots from desiccation as well as amending the soil structure and its aggregation properties result in increased soil water holding capacity and improve the overall resilience of the holobiont (Huang et al., 2022; Marasco et al., 2022). For the Atacama’s desert plants, *Cistanthe longiscapa* and *Citrullus colocynthis*, it has been reported that their survival strategy was forged through intimate interactions with associated soil bacteria and fungi (Araya et al., 2020; Procter et al., 2022). *Citrullus colocynthis* was reported to develop symbiotic interactions with plant growth-promoting bacteria such as *Acidobacteria*, *Bacterioidetes*, and *Actinobacteria* for nitrogen, sulfur, and carbon cycles, as well as for the solubilization of phosphate and the synthesis of indole-2-acetic acid and siderophores (Procter et al., 2022). The four native Saudi Arabian desert plants, *Zygophyllum simplex*, *Panicum turgidum*, *Euphorbia granulate*, and *Tribulus terrestris*, harbor bacterial strains that exhibit distinct biochemical pathways regarding nutrient uptake and survival under stress conditions (Eida et al., 2018). Recently, Abumaali et al. (2023a,b) stated that Qatari wild date palm (*Phoenix sylvestris*) displayed specific and unique bacterial operational taxonomic units (OTUs) that could improve date palm tolerance to salinity and drought. Indeed, the rhizospheric core microbiome from arid regions may improve the ability of date palm to withstand harsh environmental conditions by promoting microbe-induced systemic tolerance. To cope with abiotic stress and low organic carbon, microbes engage a multitude of direct and indirect mechanisms to support plants (Figure 1; Mohanty et al., 2021). Direct mechanisms involve the increase of vital nutrient acquisition (e.g., N, P, and Fe) (Anli et al., 2020), the accumulation of osmolytes that impart drought tolerance in plants (e.g., soluble sugars, proline, glycine, organic acids, Huang et al., 2014), the production of exopolysaccharide (Rolli et al., 2015), the regulation of phytohormone levels including auxin, gibberellin, and cytokinin, and particularly the 1-aminocyclopropane-1-carboxylate (ACC) deaminase to reduce the ethylene level in roots (Lau et al., 2022), and the induction of stress-responsive genes (e.g., *NCED*, *P5CS*) (Poudel et al., 2021). Indirect mechanisms encompass actions where microbes enhance plants’ resilience by improving soil characteristics (Ait-El-Mokhtar et al., 2020), maximizing the total area of the root, resulting in improved nutrient and water absorption (Ngumbi and Kloepper, 2016), or suppressing pathogens that may exacerbate stress conditions (Poudel et al., 2021). Through these intricate interactions, microbes play a key role in bolstering plant resilience and enabling them to thrive in challenging environments.

Next, to the advantageous ecological and protective services offered by desert plant-associated microorganisms (Köberl et al., 2016; Shilev et al., 2019; Karray et al., 2020; Gargouri et al., 2021b), few microbial profiling studies have been carried out on the rhizospheres and root systems of date palm, the iconic oasis keystone (Chebaane et al., 2020; Gagou et al., 2023; Abumaali et al., 2023a). Moreover, although high-throughput sequencing technology provides excellent opportunities for the investigation of microbiomes, studies on date palm microbiomes remain scarce.

In accordance with the available data, date palm rhizosphere soil and root systems shelter a reservoir of beneficial symbiotic microorganisms that positively regulate its homeostasis (Ferjani et al., 2015). Mosqueira et al. (2019) carried out a broad survey of bacterial diversity associated with date palm grown across the Sahara desert in Tunisia. They identified two major endophytic bacterial phyla, *Gammaproteobacteria* and *Alphaproteobacteria,* known to perform ecological functions of biopromotion and biofertilization in harsh environments. Shamim et al. (2022) demonstrated that *Micromonospora* and *Mycobacterium* bacterial taxa were effective in alleviating salinity stress when date palms were irrigated with saline water. Cherif et al. (2015) further showed that *Gammaproteobacteria*, a class of endophytic bacteria isolated from date palm, was also effective in improving plant drought tolerance. Using pyrosequencing, Yaish et al. (2016) revealed that the composition of endophytic bacterial and fungal communities in *P. dactylifera* differs according to the concentration of salt in the irrigation water.

Up-to-date, there are few reports addressing date palm microbial profiling (Ferjani et al., 2015; Yaish et al., 2016; Mosqueira et al., 2019; Chebaane et al., 2020; Al-busaidi et al., 2022; Shamim et al., 2022). Few of them go deeper beyond the species identification level. Consequently, advanced technologies, such as high-throughput sequencing, have become highly recommended to be able to characterize in depth the rhizosphere and endophytic microbiota of *P. dactylifera*, which would further contribute to dissecting more beneficial microbial taxa and better understanding their role in enhancing date palm stress mitigation.

## 3 Beneficial contributions of the use of date palm cultivable microorganisms to promote sustainable phœniciculture

The rhizosphere and endosphere of arid land habitats offer a valuable reservoir of biomolecules with fertilizing and biocontrol properties against a large spectrum of biotic and abiotic constraints (Alsharif et al., 2020). They feature a wide diversity of plant growth-promoting (PGP) microbial communities involved in vital processes, exchanging services for niches and nutrients, ultimately resulting in a win–win and high-performance partnership with the plant partner (Vacheron et al., 2013; Soussi et al., 2016). Consequently, they are regarded as potential and pertinent candidates to substitute conventional fertilizers and pesticides. This would promote food security and the sustainability of food production systems (Gargouri et al., 2021b; Ben Zineb et al., 2022).

The recent overview by Alsharif et al. (2020) on the diversity of desert plant rhizosphere microbiomes, including the latest findings and applications, reported that desert PGP microorganisms are genetically better equipped to adapt to harsh environments than those evolving in non-arid soils. Furthermore, many research teams were focusing on studying arid land-associated microbial communities to explore their beneficial agronomical contributions following their inoculation with cash crops, such as wheat (Singh and Jha, 2016), cowpea (Minaxi et al., 2012), Salicornia (Marasco et al., 2016), and date palm (Anli et al., 2020; Chebaane et al., 2020). Among the available data, a consensus emerges on the advantageous contribution provided by the inoculation of PGP bacteria on date palm to better adapt to abiotic stresses (Figure 1; Table 1).

**Table 1:** Studies depicting previously evaluated inoculation potential on date palm plants.

Stress type	Inoculation methods	Microbes used		Amendment	Response	References
		PGP (bacteria/fungi)	AMF			
Drought stress						
25, 75% FC	Rhizosphere inoculation (fresh corn root fragments and spores)	–	Autochthonous AMF consortium	Rock phosphate + local compost	Improvement of leaf number, stomatal conductance, chlorophyll fluorescence, and pigment content. Enhancement of total soluble sugar content. Decrease of hydrogen peroxide (H_2_O_2_). Improvement of soil traits.	Akensous et al. (2023)
Water regimes: 32 L/h for well-watered and 16 L/h for drought stress	Rhizosphere inoculation (fresh corn root fragments and spores)	Indigenous PGP bacteria (from the rhizosphere of palm groves)	Aoufous consortium	Organic waste-based compost	Improvement of plant biomass. Rise of phosphorus uptake, total soluble sugar, and protein content. Boost plant–water relationship. Decrease in malondialdehyde (MDA) and H_2_O_2_. Improvement of organic matter, soil phosphorus, and glomalin content.	Akensous et al. (2022a)
25, 75% FC	Fresh inoculum (roots and substrate containing spores) + soil drenching	Four PGP bacteria (from the rhizosphere of palm groves)	Exogenous AMF *Rhizoglomus irregulare* Indigenous AMF *Glomus* sp. *Sclerocystis* sp., and *Acaulospora* sp.	Grass/green waste-based compost	Enhancement of plant growth and physiological parameters. Enhancement of leaf water potential, electrical conductivity, organic matter, and total organic carbon. Improvement of N and P content. Increase in sugar and protein content. Decrease in MDA and H_2_O_2_.	Anli et al. (2020, 2021)
25, 50, 75, 100% FC	Fresh inoculum (roots and substrate containing spores) + soil drenching	*Bacillus* S48	28 species from the rhizosphere of palm grove	–	Improvement of the leaf’s relative water content. Enhancement of proline content. Decrease of the antioxidant defensive machinery: superoxide dismutase (SOD), catalase (CAT), glutathione peroxidase (POX), and glutathione S-transferase. Enhancement of electrical conductivity.	Harkousse et al. (2021)
25, 75%, FC	Fresh mycorrhizal barley root fragments, spores, and hyphae	–	Exogenous AMF *G. monosporus*, *G. Clarum*, and *G. deserticola* Indigenous AMF *Glomus* sp., *Sclerocystis* sp., and *Acaulospora* sp.	–	Increasing the number and area of date palm leaves. Higher relative water content (RWC). Improving the mineral nutrition of date palms (higher levels of P, Ca, Mg, K, and Mn). Increase of POX and polyphenol oxidase (PPO) enzyme activities.	Meddich et al. (2018), Meddich (2021)
25, 75% FC	Soil with alfalfa fresh root fragments, spores, and hyphae	–	Native AMF consortium *Rhizophagus intraradices* *Funneliformis mosseae*	–	Increasing the shoot height and biomass. Enhancement of the RWC. Increasing cell wall elasticity to maintain high RWC in leaves without lowering leaf water potential under stressful conditions.	Baslam et al. (2014)
25, 75% FC	Soil from trap cultures containing spores, hyphae, and mycorrhizal root fragments	–	*Glomus clarum*, *G deserticola*, and *G monosporus*	–	Accumulation of K^+^, Ca^2+^, Mg^2+^, and P in leaves Enhancing shoot dry weight	Faghire et al. (2010)
Long-term drought stress 25% FC	Spores conserved in sterile soil + fresh mycorrhizal barley root fragments, spores, and hyphae	–	*Rhizophagus intraradices*, *Funneliformis mosseae*	–	Alleviation of the detrimental effect of drought on growth performance Alleviation of H_2_O_2_ and MDA accumulation Improvement of antioxidant enzyme activities: CAT, SOD, ascorbate peroxidase (APX), and guaiacol peroxidase (G-POD). Decrease of oxidative damage and increase of proteins and soluble sugar contents.	Benhiba et al. (2015)
Salt stress						
Up to 7.6 dS m^−1^.	Trap cultures with date palm and common millet seeds	–	*Albahypha drummondii*, *Dominikia disticha*, *Funneliformis coronatus*, and *Rhizoglomus irregulare*	–	Positive correlation between soil salinity and easily extractable glomalin-related soil protein and spore density.	Chebaane et al. (2020)
240 mM NaCl	Fresh mycorrhizal barley root fragments, spores, and hyphae	–	Exogenous AMF *G. monosporus*, *G. Clarum*, and *G. deserticola* Indigenous AMF *Glomus* sp., *Sclerocystis* sp., and *Acaulospora* sp.	–	Greater AMF colonization of date palm roots. High stomatal conductance. High level of RWC and leaf water potential under salt stress compared to control plants.	Meddich et al. (2018)
0, 50, 100, and 200 mM NaCl	Seed coating application	*Bacillus* and *Enterobacter*	–	–	Production of 1-aminocyclopropane-1-carboxylic acid (ACC) altering plant ethylene levels. Production of ammonia. Solubilization of phosphate ion (PO_4_^3−^) and zinc ion (Zn^2+^). Enhancement of seedling root elongation.	Yaish et al. (2015)
0, 10, and 20 g·L^−1^ NaCl	Fresh mycorrhizal corn root fragments	–	Autochthonous AMF Exogenous AMF	–	Improvement of growth parameters. Enhancement of antioxidant enzyme activities.	Outamamat et al. (2021)
0 and 240 mM NaCl	Solid substrate (roots and substrate containing spores)	–	Indigenous AMF: *Glomus* sp., *Sclerocystis* sp., and *Acaulospora* sp.	–	Improvement of physiological parameters through elevating stomatal conductance, photosynthetic efficiency, and leaf water potential. Delayed salt stress effects on nutrient uptakes. Amelioration of P, K as well as Ca content. Decrease in MDA and H_2_O_2_ Rise in SOD, CAT, POX as well as APX activities	Ait-El-Mokhtar et al. (2019, 2021)
0 and 240 mM NaCl	–	–	Autochthonous AMF consortium	Green waste compost	Increase in P and Ca^2+^ uptake, chlorophyll content, relative water content, stomatal conductance, antioxidant enzymatic activities (superoxide dismutase, ascorbate peroxidase, catalase) Decrease in lipid peroxidation and H_2_O_2_ content.	Ait-El-Mokhtar et al. (2020, 2022)
0, 120, and 240 mM NaCl	Fresh inoculum (roots and substrate containing spores) + spraying closely to the roots	PGP bacteria isolated from DP rhizospheric soil	Native AMF: *Glomus* sp., *Sclerocystis* sp., and *Acaulospora* sp. Exotic AMF *Rhizophagus irregularis*	Green waste-based compost	Increasing plant growth: plant height; leaf number; and fresh and dry weights of shoots and roots. Accumulation of osmotic adjustment compounds and antioxidant enzyme activity Increasing total chlorophylls, carotenoids, and chlorophyll a and b content. Increasing soluble sugars and protein	Toubali et al. (2020)

FC, field capacity; DP, date palm; AMF, arbuscular mycorrhiza fungi; PGP, plant growth promotion; RWC, relative water content.

### 3.1 Contribution of PGP microorganisms to abiotic stress mitigation in date palm

#### 3.1.1 Salinity

Producing dates with an economically profitable yield and competitive quality under the constraints of continuously increasing salinity remains a difficult challenge to overcome. Selection of tolerant date palms to salinity was addressed by means of *in vitro* culture (Roy et al., 2014), given that working directly in the soil makes the task difficult in arid and hyper-arid regions affected by salinity (Al Kharusi et al., 2019). Studies indicate that close inoculation of date palm with PGP bacteria (Figure 1) could reduce oxidative stress, directly or indirectly, for example, via the accumulation of osmolytes or by the production of hormones capable of modulating the plant’s response (De Zelicourt et al., 2013; Hazzouri et al., 2020). Yaish et al. (2015) reported that date palm endophytic bacteria synthesize 1-aminocyclopropane-1-carboxylic acid (ACC) deaminase capable of cleaving part of ACC (a precursor in the ethylene biosynthesis pathway) induced by salt stress and causing inhibition of root elongation (Lau et al., 2022). This molecule can thus play a role in promoting the growth and development of the date palm in saline environments.

Furthermore, date palm nutrient utilization under salinity constraints can be improved when associated with mycorrhiza. This symbiotic relationship may enhance their yield by improving absorption of soil nutrients through fungal hyphae extension in the rhizosphere area, thereby increasing surface uptake (Akensous et al., 2022b). Accordingly, Meddich et al. (2018) noticed a consistent increase of Ca, P, K, Mg, and Mn when date palm seedlings, inoculated with arbuscular mycorrhizal fungi (AMF), were subjected to different stresses. Moreover, the inoculation of date palm plantlets with a consortium made of AMF and a mixture of *Glomus* sp., *Sclerocystis* sp., and *Acaulospora* sp. improved the physiological responses of the stressed plants (photosynthetic efficiency, leaf water potential, and stomata conductance). The enhancement of photosynthetic capacity resulted in a higher capacity of gas exchange, a better efficiency of the photosystem II (PS II), and a more efficient regulation of the energy flow between the photochemical reactions and the non-photochemical reactions (Ait-El-Mokhtar et al., 2019, 2022). More interestingly, the strains of AMF from Tunisian oasis ecosystems increased the fraction of easily extractable glomalin-related soil protein (EE-GRSP), suggesting that the AMF undergo a survival mode to mitigate the negative effects of salt stress for themselves as well as for their date palm host plants (Chebaane et al., 2020).

#### 3.1.2 Drought

In addition to strengthening date palm trees’ resilience to salinity, arid land’s microorganisms employ different pathways to counter the negative consequences of drought, mainly in young date palm plantations (Hazzouri et al., 2020). They induce systemic tolerance by triggering a series of biochemical and physiological responses. In this respect, Harkousse et al. (2021) reported that inoculation with a consortium composed of 28 species of rhizosphere AMF, collected from an oasis palm grove, improved the relative water content of the leaves of stressed date palm plants and increased their proline content, a fundamental osmoregulation solute (Liang et al., 2013). Next to this, Anli et al. (2020) reported that the co-inoculation with plant growth-promoting rhizobacteria and AMF (composed of *Sclerocystis* sp., *Acaulospora* sp., and *Glomus* sp.) increased date palm protein and soluble sugar contents and boosted the antioxidant defense activity. Date palm plants inoculated and subjected to water stress responded with an increase in their water potential and water content, which ensured the maintenance of physiological turgor levels. It should be noted that the accumulation of osmolytes and the strengthening of antioxidant power can contribute to osmotic regulation, the maintenance of cellular turgor, the preservation of cellular structures, and the traps of reactive oxygen species [e.g., hydrogen peroxide, H_2_O_2_, and malondialdehyde (MDA)]. This likely resembles a primary avoidance strategy developed by date palms inoculated in response to water stress. Furthermore, the inoculation of date palm roots, indoor and outdoor, activates hormonal biosynthesis (ABA, for example) to ensure acceptable levels of stomatal and photosynthetic activities (Meddich et al., 2021). Indeed, inoculated date palms responded with an increase in the elasticity of their leaf cell walls and modified the redistribution of water between the symplastic and the apoplastic compartments (Baslam et al., 2014). Therefore, this could serve as an alternative strategy to survive water stress through corrective responses at the level of vital physiological attributes such as relative water content (RWC), electrolyte loss/leakage (EL), and stomatal conductance.

Overall, the inoculation with microorganisms from the date palm rhizosphere could serve as an integrated approach to improve date palm defenses and mitigate the negative effects imposed by salt and water stresses and by pathogens. Such inoculation could serve as an effective means to improve their growth and productivity under future climate change scenarios.

## 4 Current challenges limiting date palm biofertilizer efficiency in arid conditions and potential solutions

Cultivated date palm microorganisms’ inoculum has been used to boost date palm resistance to abiotic and biotic stresses and would represent potential substitutes for conventional pest control products (Meddich et al., 2018; Omomowo et al., 2023). Nevertheless, they remain facing efficiency, technical, and sustainability challenges (Qiu et al., 2019). To reach an acceptable efficiency level on date palm, these beneficial microbes must overcome key steps: establishment, survival, colonization, and interaction with the host tree.

### 4.1 Soil-related factors

Date palms are typically grown in arid regions where soils are often coarse-textured and calcareous, deficient in nutrients and organic matter, and where the pH is rather alkaline (Alotaibi et al., 2023). Therefore, the availability of nutrients and the effectiveness of fertilizers, particularly phosphorus, can affect their development. Indeed, phosphorus represents the macronutrient most sensitive to soil pH, and its availability in alkaline and calcareous soils is made low in particular by the presence of Ca^2+^, whose precipitation and retention power are rather high (Ben Zineb et al., 2019b). The use of slow-release phosphorus fertilizer, such as rock phosphate, might be a potential solution (Ben Zineb et al., 2022). Moreover, to reduce the adverse effects of limestone, phosphate-solubilizing microorganisms’ amendments are recommended. Yet, interactions between phosphate-solubilizing microbes with intrinsic soil properties (humidity, water and nutrient availability, temperature, pH, etc.) and date palm exudates must also be taken into account (Uroz et al., 2019; Fitzpatrick et al., 2020). Additionally, current strategies implemented in soil management are rather inappropriate (Almadini et al., 2021), as they are heavily dependent on fertilizers produced by the chemical industry, which are often harmful to soil microorganisms. Indeed, chemical fertilizers might be partially immobilized right after their application, which induces lower root colonization and thereby limited inoculation efficiency. In this respect, El Hilali et al. (2022) found reduced levels of mycorrhizal root colonization in date palms receiving synthetic fertilizers.

### 4.2 Plant-related factors

In the date palm multiplication process, the transition from the laboratory to the field, acclimation, is one of the most critical stages, as it represents the transition from an assisted or autotrophic lifestyle to an autonomous/self-sufficient or heterotrophic mode (Nazir et al., 2015; Solangi et al., 2022). Indeed, after *in vitro* cultivation, the plantlets are extracted from their synthetic cultivation medium and transferred into soil and acclimatized in a less controlled environment, where they have to adapt to survive (different light, less humidity, different atmosphere, different nutrients, and substrate) (Hassan, 2017; Saadaoui et al., 2019). In addition, plants can modify, directly or indirectly, the habitat of the rhizosphere, notably by rhizodeposits, which end up modifying the surrounding conditions of the roots. Therefore, research work remains necessary to identify the optimal physiological stage of date palm for inoculation with PGP microorganisms in order to obtain maximum benefit.

### 4.3 Inoculant-related factors

Inoculant formulation is a critical aspect and should be optimized to allow high competition and survivability of the inoculum under severe environmental conditions. The exogenous microorganisms have to overcome colonization issues and establish a symbiotic and beneficial environment for both entities (Ferjani et al., 2015). Indeed, the persistence of the inoculated microorganisms can be promoted via the use of consortia composed of resistant PGP microorganisms (e.g., engineered microbial communities, SynComs) rather than monocultures containing single selected strains in order to improve the survival rates and the physiological activity of the microbial inoculum (Uroz et al., 2019; Fitzpatrick et al., 2020). In this respect, Anli et al. (2020) reported that a close-up inoculation of date palm with indigenous PGP bacteria increased the AMF root system infection under drought stress, probably because the inoculated bacteria enhanced the AMF multiplication and activity (Ben Zineb et al., 2022).

Generally, inoculant development mainly focuses on genetic and PGP traits and often neglects ecological traits that are critical to the success of inoculations (Kaminsky et al., 2019). The release of exotic species into the rhizosphere of date palms risks disrupting their ecological balance, which would make indigenous communities less competitive and therefore more vulnerable compared to exogenous species. Therefore, the isolation and screening of pre-adapted dryland microorganisms should take into consideration both PGP and ecological criteria, which means strains that are both functional and have increased environmental adaptation potentials (Mefteh et al., 2018; Ben Zineb et al., 2019a; Toubali et al., 2020; Harkousse et al., 2021). Furthermore, due to sampling constraints, extensive niche specialization, and the low adaptability of conventional cultural practices, many date palm microorganisms were neglected in terms of cultivation and characterization in the laboratory (Bull et al., 2016). Consequently, the creation of complete collections of strains via systematic culturomics, diversifying culture conditions, and taking advantage of high-throughput sequencing should improve our understanding of the diversity of cultivable rhizosphere microbiomes of the date palm (Matar and Bilen, 2022; Li et al., 2023).

Additionally, the formulation of the inoculant has to support, at the same time, microbial growth and the protection of viable cells in order to trigger an efficient response in date palm (Bashan et al., 2016). An inoculum formulation using varied and innovative technologies should be tested (microencapsulation, nanotechnology, etc.) to increase the efficiency of inoculum application (Kragh et al., 2018). Furthermore, it is recommended that date palm pre-adapted inoculum and/or selected compounds of prebiotics, such as phytoalexin and triterpenes, be used in order to favor microbes of interest (Pantigoso et al., 2023). Finally, a multiple approach combining conventional pathogenicity tests, targeting non-target organisms, and genomics should be implemented before field dissemination of the inoculum (Qiu et al., 2019).

AMF counts among the most represented, oldest, most widespread, and most important symbioses on Earth that contribute to feeding the world. They associate with an estimate of 72% of land plants and can deliver up to 90% of the total plant phosphorus, making these microorganisms a focal point of attention for many biotech companies. AMF inoculum production remains mainly limited to *in vivo* systems (e.g., greenhouse), which are often inexpensive and suitable for large-scale production with densities reaching 80–100 propagules per cm^3^ of substrate (Gianinazzi et al., 2002). Yet, these cultivation systems are not exempt from contaminants and may require large spaces. Their cultivation under *in vitro* conditions using whole plants or their organs is a promising alternative to producing high-quality inoculum that is free of any contaminants and requires limited space. Still, *in vitro* production yields do not reach the level of *in vivo* systems; they are costly and restricted to a few species (Ijdo et al., 2011). Therefore, there is an urgent need to propose novel *in vitro* cultivation systems providing clean, safe, and robust spore date palm inoculum, produced at high densities and with reduced costs (Gargouri et al., 2021a).

At the application level, administration practices must favor the protection of the inoculated microbes against environmental stresses. Thus, the inoculation technique is decisive for the success of the inoculation.

### 4.4 Stress-related factors

The date palm is in constant challenge against lack of water, rising salinity, extreme temperatures, degradation and loss of soil fertility, pests, and diseases. The combination of multiple stresses of biotic and abiotic nature (high salinity, accentuated drought, high temperatures, pathogens, etc.) or abiotic stresses alone (salinity stress, water stress, or thermal stress) could have more harmful effects on the survival of the date palm (Safronov et al., 2017; Khan et al., 2020). In this respect, Meddich et al. (2021) reported that the effect of drought induces more severe damage when the plant is at the same time infected with *Fusarium oxysporum* f. sp. albedinis (Foa). High temperatures also make plants more vulnerable to diseases and contribute to the emergence of more virulent pathogens (Ali et al., 2023). Khan et al. (2020) revealed a more significant diminution of shoot elongation when date palms were simultaneously exposed to a combined stress composed of salt and cadmium than when they were exposed to a single stress (NaCl or Cd). In fact, when cadmium (Cd) interacts with NaCl, there is the formation of Cd-Cl complexes, which act as stimulators of Cd uptake by the plant. However, studies dealing with the effect of multiple stresses (more than two) on tolerant microbiota to monitor date palm cultivar responses are still missing. Consequently, systematic studies are pivotal to understanding the tripartite interactions involving date palm trees, stress-tolerant microbiota, and multi-abiotic stresses (Anderegg et al., 2015). Ultimately, the development of an approach for beneficial microbiota tolerant to multiple stresses will make it possible to better understand the behavior of the date palm with respect to climate change.

## 5 Desert plants microbes’ inoculation approach to promote sustainable date palm production

### 5.1 Literature search strategy

We collected and analyzed the available literature (mainly international peer-reviewed studies) published in recent years from 2010 up to May 2023 based on the search engines of Web of Science, ScienceDirect, and Google Scholar, using the following keywords: “Qatar and date palm,” “Qatar and microbiome,” “Qatar and soil microbial communities,” “Qatar and soil bacterial communities,” “Qatar and soil fungal communities,” “Qatar and soil mycorrhizae.” This query led to insufficient results because of the lack of studies on the investigated topics, particularly those studying microbial ecology, community structure, and their interactions with date palm plants.

### 5.2 Overview and future research directions

In the GCC, where water scarcity and desert conditions pose significant challenges to agriculture (Al-Khateeb et al., 2020), date palm trees have been of keen research interest as they are among the main agricultural sectors concerned by the sustainability issue (Al Nabil, 2021; Karanisa et al., 2021). Despite multiple programs aiming at the rehabilitation and rescue of palm groves (Meddich et al., 2018), the soil microbiota associated with the date palm is poorly represented in global microbial databases. Moreover, there are still no detailed reports based on modern science on the microbiome of wild plant species in the GCC soils (Al-Thani and Yasseen, 2021; Abumaali et al., 2023a).

Although ancestors of wild date palm populations exist in remote areas of the GCC region and have been shown to be quite different from those found in Africa and the Middle East (Gros-Balthazard et al., 2018), only a limited number of publications report on wild date palm and desert plants or the inoculation of cultivated date palm (Abumaali et al., 2023a). It is obvious that the restoration of any ecosystem needs to integrate different components and data available on the ecosystem in question, in particular the microorganisms associated with the local plants (Anli et al., 2020).

**Figure 2:**
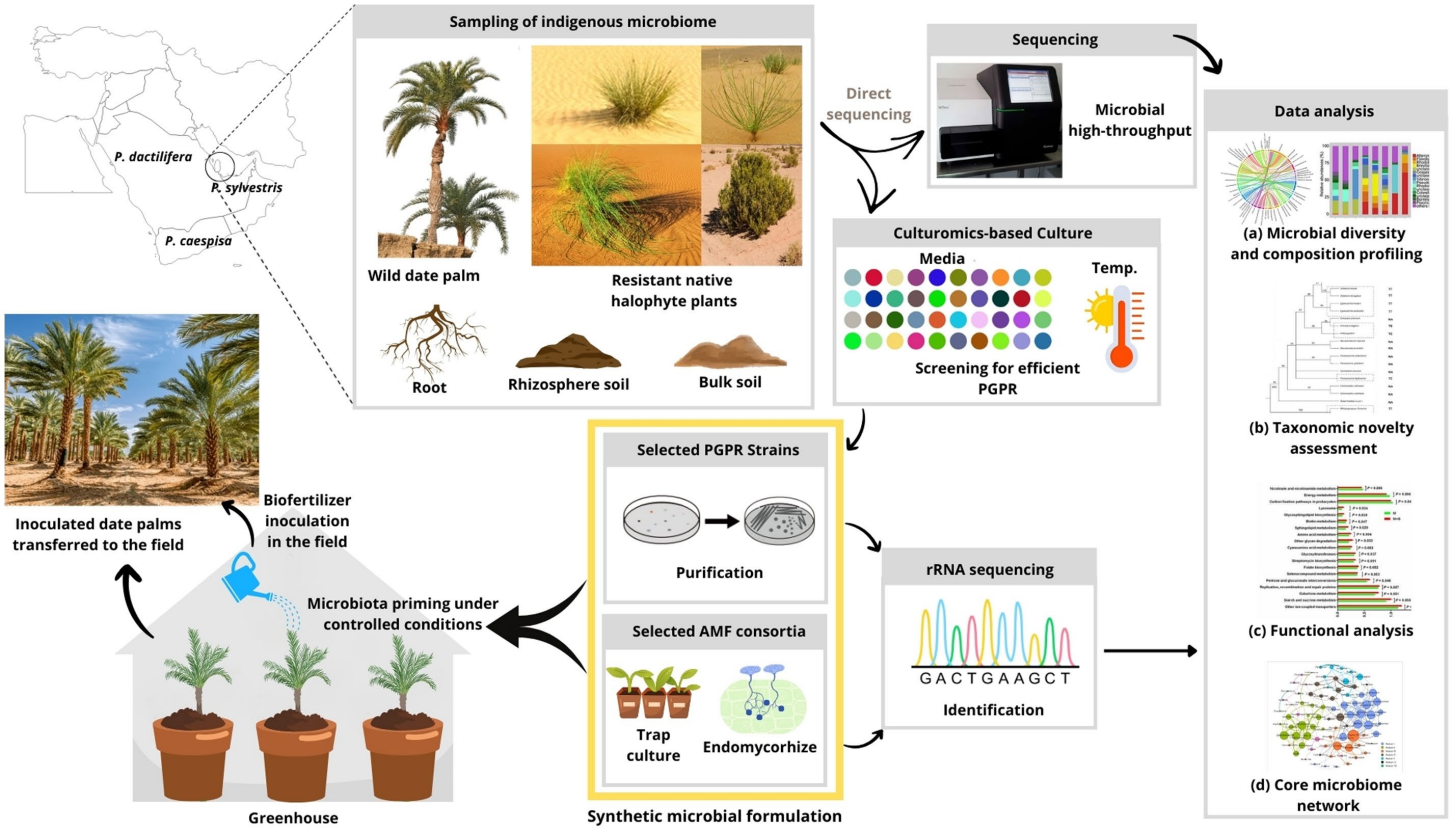
Road map of future studies that will further promote the sustainability of date palm in the GCC. Based on the research question of interest, the concept of a synthetic microbial community generated from indigenous microbes screened from the autochthone wild date palm populations (*P. sylvestris*) and native desertic plants is necessary to pave the path for beneficial agriculture practices. The application of culturomics technology and microbial high-throughput sequencing to determine (a) the microbial diversity and composition profiling, (b) the taxonomic novelty assessment, (c) the microbial functional analysis, and (d) the core microbiome network of the native plants. This knowledge should help guide next-generation field applications to promote the sustainability of date palm cultivation.

Accordingly, in order to enhance date palm resilience to climate change and promote sustainable cultivation and production of dates in Qatar, a number of research priorities were identified: (i) To aid in understanding how date palm–microbe interactions would help them resist in the desert environmental stresses, further investigation is required to have a more holistic perception regarding date palm root-associated microbiome, especially wild date palm, as suggested in Figure 2. The use of modern high-throughput sequencing should improve the characterization of the composition and performance of the rhizospheric and endophytic microbiota of the date palm to be able to exploit and valorize it optimally. (ii) Given the scarcity of data on desert microbiome and the effect of their application in date palm oases in Qatar, it’s important to note that research and experimentation specific to autochthone microbes would be necessary to determine their most effective synthetic combination and optimal screening and application methods for a profitable cultivation of date palm in the area (Figure 2), paving the path for beneficial agricultural applications (Al-Yahya’ei et al., 2011). (iii) Future research programs using agro-ecological approaches should prioritize the maintenance and improvement of soil fertility and structure. Practices like cover plants and adapted halophytes for ecological or agricultural purposes can enhance soil organic matter and reduce erosion, leading to healthier and more productive soils. In addition, it makes sense to provide a microclimate favorable to the development of complementary underlying crops, particularly of a fodder nature (Toubali et al., 2020). Overall, recognizing the importance of integrating different ecosystem components, particularly microorganisms, for ecosystem restoration, research priorities have been identified to enhance date palm resilience in the GCC. These priorities include investigating date palm–microbe interactions, optimizing high-throughput sequencing for characterizing the microbiota, studying desert microbiomes for application in date palm cultivation, and implementing agro-ecological approaches to improve soil fertility. A holistic model, suggested in Figure 2, should be developed, involving modern and environmentally friendly agricultural technologies, to serve as a lever and catalyst for establishing sustainable agriculture and economy.

## Author contributions

AB: Conceptualization, Data curation, Writing – original draft, Writing – review & editing. ML: Writing – review & editing. AK: Writing – review & editing. HH: Writing – review & editing. TA: Writing – review & editing. HA-J: Writing – review & editing. MA: Writing – review & editing. AM: Writing – review & editing. SS: Funding acquisition, Writing – review & editing. MG: Writing – original draft, Writing – review & editing.
